# CXCR7-TAGLN2 protein complex regulates invasion and metastasis in papillary thyroid carcinoma: a potential therapeutic target

**DOI:** 10.3389/fimmu.2025.1627419

**Published:** 2025-10-15

**Authors:** Chao Zeng, Feng Wang, Yaomin Huang, Hengwei Zhang

**Affiliations:** ^1^ Department of General Surgery, the First Hospital of Lanzhou University, Lanzhou, China; ^2^ The First School of Clinical Medicine, Lanzhou University, Lanzhou, China; ^3^ Gansu Province Key Laboratory of Biological Therapy and Regenerative Medicine Transformation, Lanzhou, China

**Keywords:** papillary thyroid carcinoma, CXCR7, Tagln2, metastasis, TGF-β signaling

## Abstract

**Objective:**

To investigate the expression and clinical significance of CXCR7 and TAGLN2 in papillary thyroid carcinoma (PTC), and to explore the molecular mechanisms underlying the interaction between CXCR7 and TAGLN2 in regulating PTC invasion and metastasis.

**Methods:**

Paraffin-embedded tissue sections were obtained from 64 patients with PTC and 24 patients with nodular goiter who underwent surgical resection at The First Hospital of Lanzhou University between January 2017 and August 2020. Immunohistochemistry (IHC) was performed to assess protein expression levels of CXCR7 and TAGLN2. The associations between their expression and clinicopathological characteristics of PTC, as well as the correlation between CXCR7 and TAGLN2 expression levels, were analyzed. Human PTC cell lines were cultured *in vitro*, and co-immunoprecipitation (Co-IP) and immunofluorescence colocalization assays were conducted to evaluate the intracellular interaction between CXCR7 and TAGLN2. Lentiviral cotransfection techniques were employed to investigate the role of CXCR7 in modulating PTC cell invasion and metastasis via TAGLN2. Western blot analysis was performed to assess the levels of phosphorylated Smad2 (p-Smad2) and total Smad2.

**Results:**

IHC results demonstrated that CXCR7 and TAGLN2 expression levels were significantly elevated in PTC tissues compared to benign thyroid tissues. High expression of both proteins was significantly associated with lymph node metastasis in PTC patients, and a significant positive correlation was observed between CXCR7 and TAGLN2 expression levels. In TPC-1 and BCPAP cells, CXCR7 and TAGLN2 exhibited similar subcellular localization and physically interacted. Silencing TAGLN2 markedly reduced TPC-1 and BCPAP cell migration, while concomitant overexpression of CXCR7 reversed this inhibitory effect. Mechanistically, Western blot analysis revealed that TAGLN2 knockdown led to a substantial decrease in p-Smad2 levels, confirming TAGLN2’s contribution to TGF-β signaling activation. Furthermore, re-introducing CXCR7 into TAGLN2-silenced cells restored p-Smad2 levels, demonstrating that CXCR7 actively promotes TGF-β/Smad2 signaling.

**Conclusion:**

CXCR7 and TAGLN2 are overexpressed in PTC and correlate closely with lymph node metastasis. CXCR7 may regulate PTC cell migration and invasion through interaction with TAGLN2, primarily by activating the TGF-β/Smad2 signaling pathway. The CXCR7-TAGLN2 protein complex represents a potential novel therapeutic target for PTC.

## 1 Introduction

Thyroid cancer (TC) is the most common endocrine malignancy and is projected to become the fourth most prevalent cancer worldwide by 2030 (1). Papillary thyroid carcinoma (PTC) is the predominant histological subtype, accounting for approximately 84% of all TC cases (2). Although the prognosis for most PTC patients is favorable, 5–10% develop distant or regional metastases and may progress to radioactive iodine-refractory (RAIR) disease. The development of RAIR-PTC is frequently linked to genetic alterations affecting iodine uptake and metabolism, resulting in treatment resistance. Current targeted therapies, including BRAF and MEK inhibitors, can extend progression-free survival in RAIR-PTC patients but have limited impact on overall survival and are often associated with significant adverse effects, thereby impairing quality of life. Therefore, there is an urgent need to identify novel therapeutic targets aimed at inhibiting PTC metastasis to improve both survival outcomes and patient quality of life.

CXCR7, a recently identified atypical chemokine receptor initially classified as an orphan receptor, predominantly binds stromal cell-derived factor 1 (SDF-1, also known as CXCL12) *in vivo*. This interaction activates intracellular signaling pathways, including the AKT and MAPK pathways, via β-arrestin-mediated signaling, thereby contributing to tumor progression and modulation of the tumor microenvironment (3, 4). CXCR7 is overexpressed in various human malignancies, including lung, colorectal, hepatic, prostate, and gastric cancers, and its expression is associated with aggressive phenotypes such as enhanced proliferation, migration, and invasion (5–9). Our previous studies have shown that CXCR7 is significantly upregulated in PTC tissues compared with adjacent non-tumor tissues, and this overexpression is correlated with lymph node metastasis (10). Furthermore, silencing CXCR7 inhibits PTC cell growth, proliferation, invasion, and angiogenesis, while promoting apoptosis (11, 12). These findings indicate that CXCR7 plays a critical role in the development and metastasis of PTC. However, the precise molecular mechanisms underlying CXCR7-mediated regulation of PTC pathogenesis remain largely unclear.

TAGLN2 (Transgelin-2), an actin-binding protein, plays a pivotal role in cytoskeletal remodeling through its interaction with actin filaments. Elevated expression of TAGLN2 has been reported in multiple malignancies, including colorectal cancer (13), bladder cancer (14), lung cancer (15), cervical squamous cell carcinoma (16), and breast cancer (17). Notably, Wang et al. (18) demonstrated that high TAGLN2 expression in PTC is positively associated with lymph node metastasis, and that TAGLN2 overexpression promotes PTC cell proliferation, migration, invasion, and angiogenesis. Additionally, our proteomic analysis of a CXCR7-overexpressing PTC cell line revealed significantly increased levels of TAGLN2 (19). These observations suggest that CXCR7 may be involved in TAGLN2-mediated PTC metastasis. Nevertheless, the exact mechanisms by which CXCR7 interacts with TAGLN2 to regulate PTC invasion and metastasis remain undefined.

This study aims to evaluate the immunohistochemical expression of CXCR7 and TAGLN2 in PTC tissues (experimental group) and nodular goiter tissues (control group), investigate their associations with clinicopathological features of PTC, and assess the correlation between their expression levels. Subsequently, PTC cell lines were cultured to examine the intracellular interaction between CXCR7 and TAGLN2 proteins using immunofluorescence co-localization and co-immunoprecipitation assays. Finally, lentiviral co-transfection was employed to silence TAGLN2 and simultaneously overexpress CXCR7, followed by assessment of PTC cell migration and invasion capacities, to elucidate the molecular mechanism by which CXCR7 regulates PTC invasion and metastasis through interaction with TAGLN2.

## 2 Materials and methods

### 2.1 Clinical tissue samples and pathological data

Formalin-fixed, paraffin-embedded tissue specimens were collected from 64 patients with papillary thyroid carcinoma (experimental group) and 24 patients with nodular goiter (control group) who underwent surgical resection at the First Hospital of Lanzhou University between January 2017 and August 2020. Tissue sections were prepared for immunohistochemical analysis. The use of all specimens was approved by the Ethics Committee of the First Hospital of Lanzhou University, and complete clinical data were available for all cases.

### 2.2 Immunohistochemical staining (SP method)

The tissue samples were embedded in paraffin and sectioned at a thickness of 4 μm. Following overnight baking of the pathological sections, deparaffinization was performed sequentially in xylene, followed by rehydration through a graded ethanol series. Antigen retrieval was achieved by boiling the sections in sodium citrate buffer for 5 minutes. Endogenous peroxidase activity was blocked with 3% hydrogen peroxide. To minimize non-specific binding, sections were incubated with 10% goat serum albumin for 1 hour at room temperature, then incubated overnight at 4°C with primary antibodies against CXCR7 (1:100) and TAGLN2 (1:200). Subsequently, sections were sequentially incubated with biotinylated secondary antibody (goat anti-rabbit IgG) and horseradish peroxidase-conjugated streptavidin, each at 37°C for 30 minutes. Between each incubation step, sections were washed three times with PBS (5 minutes per wash). Diaminobenzidine (DAB) was used as the chromogen for signal detection, followed by counterstaining of nuclei with hematoxylin. Finally, sections were dehydrated through a graded alcohol series and mounted.

Quantitative analysis was performed using the Immunoreactivity Score (IRS) system. The IRS was calculated as follows: IRS = Staining Intensity (SI) × Percentage of Positive Cells (PP). SI was scored on a four-point scale: 0 (negative), 1 (weakly positive), 2 (moderately positive), and 3 (strongly positive). PP was categorized as: 0 (negative), 1 (1–25%), 2 (26–50%), 3 (51–75%), and 4 (76–100%). For each sample, three representative fields from different regions were randomly selected for IRS evaluation, and the mean IRS value was recorded as the final score. An IRS < 3 was defined as low expression, whereas an IRS ≥ 3 was considered high expression.

### 2.3 Cell culture and reagents

The human papillary thyroid carcinoma cell line TPC-1 and BCPAP were obtained from Wuhan Procell Life Science and Technology Co., Ltd. Cells were maintained in RPMI-1640 medium supplemented with 10% fetal bovine serum and 1% penicillin-streptomycin solution. All media and supplements were purchased from Biological Industries (Israel).

Primary antibodies used were as follows: rabbit polyclonal anti-phospho-Smad2 (p-Smad2) (CST, USA, 138D4), rabbit polyclonal anti-Smad2 (CST, USA, D43B4), rabbit polyclonal anti-GAPDH (Bioworld, USA, AP0063), rabbit polyclonal anti-CXCR7 (GeneTex, USA; GTX100027), rabbit polyclonal anti-TAGLN2 (Abcam, USA; ab121146), and mouse monoclonal anti-TAGLN2 (Proteintech, USA; 60044-1-Ig). Protein A/G PLUS-Agarose was acquired from Santa Cruz Biotechnology (USA), and Gαppen cyclic peptide-TRITC was purchased from Sigma-Aldrich (USA).

### 2.4 Co-localization by immunofluorescence

Cells in the logarithmic growth phase were dissociated into single-cell suspensions and seeded onto 96-well glass-bottom plates. After reaching confluence, cells were fixed with 4% paraformaldehyde, permeabilized with 0.2% Triton X-100, and blocked with PBS containing 5% bovine serum albumin (BSA). Primary antibodies (CXCR7, 1:500; TAGLN2, 2 μg/mL) were applied and incubated overnight at 4°C. Following washing, cells were incubated with green fluorescent dye-labeled secondary antibody at 37°C for 45 minutes in the dark. Nuclei were counterstained with DAPI, and filamentous actin was labeled with phalloidin conjugated to red fluorophore (5 μg/mL) for 40 minutes at room temperature in the dark. After each incubation, cells were washed thoroughly with PBS. Fluorescence imaging was performed using a fluorescence microscope.

### 2.5 Co-immunoprecipitation experiment

According to the manufacturer’s instructions, cells in the logarithmic growth phase were washed with pre-cooled phosphate-buffered saline (PBS) and lysed on ice using IP lysis buffer. The supernatant was collected, and a small aliquot was reserved for Western blot analysis. The remaining lysate was incubated with 1 μg of CXCR7 antibody at 4°C overnight. Pre-treated protein A/G agarose beads were added to bind the antibody-protein complex, followed by gentle shaking incubation at 4 °C for 2 h. The agarose beads were pelleted by centrifugation, washed with lysis buffer, and then boiled in 5× SDS loading buffer for denaturation. Protein concentration was determined using the BCA assay. Proteins were separated by SDS-PAGE and transferred onto polyvinylidene fluoride (PVDF) membranes via wet transfer. Membranes were blocked with 5% skim milk and incubated with primary antibodies against TAGLN2 (1:1000) and CXCR7 (1:1000) at 4°C overnight. After washing with TBST, corresponding secondary antibodies were applied, followed by additional washes. Immunoreactive bands were visualized using enhanced chemiluminescence (ECL) reagent.

### 2.6 Cell transfection and transient transfection

Short hairpin RNA (shRNA) sequences targeting TAGLN2 (5′-GTGCTATGTGAGCTCATTA-3′, knockdown [KD]) and a negative control shRNA (5′-TTCTCCGAACGTGTCACGT-3′, NC) were constructed by GeneChem (Shanghai, China). Human full-length CXCR7 complementary DNA (cDNA) was cloned into the GV341 vector (GeneChem Corp., China) for overexpression (OE), along with an overexpression negative control (5′-GGGTCAATATGTAATTTTCAGTG-3′, NC). Lentiviral particles pre-packaged with Polybrene (final concentration: 5 μg/mL) were used to transduce TPC-1 and BCPAP cells according to the manufacturer’s protocol. Based on preliminary optimization experiments balancing transduction efficiency and cell viability, the multiplicity of infection (MOI) was set to 20 for TPC-1 cells and 10 for BCBAP cells.

### 2.7 Cell migration and invasion experiments

Transwell chambers (8 μm pore size, Costar, Corning, NY, USA) placed in 24-well plates were used for both cell migration and invasion assays. For invasion assays, the upper chamber membrane was precoated with Matrigel (BD Biosciences, Cat. No. 356234; stock concentration approximately 8–12 mg/mL), diluted 1:9 in serum-free 1640 to a final concentration of approximately 0.8-1.2 mg/mL. A total of 5 × 10^4^ cells in serum-free medium were seeded into the upper chamber, while medium containing 10% fetal bovine serum (FBS) was added to the lower chamber. After 24 h of incubation, non-migrated or non-invaded cells in the upper chamber were removed. Migrated or invaded cells on the lower surface of the membrane were fixed with 4% paraformaldehyde for 20 min and stained with crystal violet for 30 min. Transmembrane cells were counted under a microscope in randomly selected fields.

### 2.8 Western blotting

Briefly, cells (TPC-1 and/or BCPAP) were lysed in RIPA buffer containing protease and phosphatase inhibitors. After centrifugation, equal amounts of protein (20-30 μg) were separated by SDS-PAGE, transferred to PVDF membranes, blocked with 5% non-fat milk, and probed with primary antibodies against p-Smad2 (1:1000), Smad2 (1:1000), and GAPDH (1:5000). Membranes were then incubated with HRP-conjugated secondary antibodies, and protein bands were visualized using ECL reagents. Band intensities were quantified using ImageJ software and normalized to the loading control (GAPDH).

### 2.9 Bioinformatics analysis

To investigate the clinical relevance and prognostic value of CXCR7 and TAGLN2 in PTC, transcriptomic and clinical data for 510 PTC cases were retrospectively acquired from The Cancer Genome Atlas (TCGA) database via the UCSC Xena Hub. Transcriptomic data, normalized to Transcripts Per Million (TPM), served as the basis for ssGSEA calculations. Specifically, ssGSEA scores for the Hallmark, Gene Ontology (GO), and Kyoto Encyclopedia of Genes and Genomes (KEGG) gene sets were computed using the GSVA package in R (version 4.4.2). Subsequently, correlation analyses were performed to assess the association between the transcript abundances of CXCR7 and TAGLN2 and their respective ssGSEA scores. Further evaluation of clinical impact involved assessing the prognostic significance of CXCR7 and TAGLN2 expression levels through Kaplan-Meier (K-M) survival analysis.

### 2.10 Statistical methods

Statistical analyses were performed using SPSS (version 23.0) for in-house data and R (version 4.4.2) for TCGA data. Fisher’s exact test was primarily used for comparisons between PTC and benign thyroid tissues, and for associations with clinicopathological features (lymph node metastasis, age, gender, tumor size, capsular infiltration, multifocality), especially when expected cell counts were low. Chi-square tests were applied where appropriate. Spearman correlation analysis assessed the relationship between CXCR7 and TAGLN2 protein expression. All association analyses reported effect sizes (Odds Ratios, ORs) with 95% Confidence Intervals (CIs). One-way ANOVA with *post-hoc* tests evaluated intergroup differences among control, silencing, and overexpression groups. Data are presented as mean ± SD. A two-tailed p<0.05 was considered significant. Transcriptomic and clinical data from the TCGA PTC database were used. Spearman correlation analyzed relationships between CXCR7/TAGLN2 gene expression and TGF-β pathway gene sets (Hallmark, GO, KEGG). Kaplan-Meier curves (dichotomized by median expression) assessed overall survival.

## 3 Results

### 3.1 CXCR7 and TAGLN2 expression levels and clinical prognostic significance in PTC

**Figure 1:**
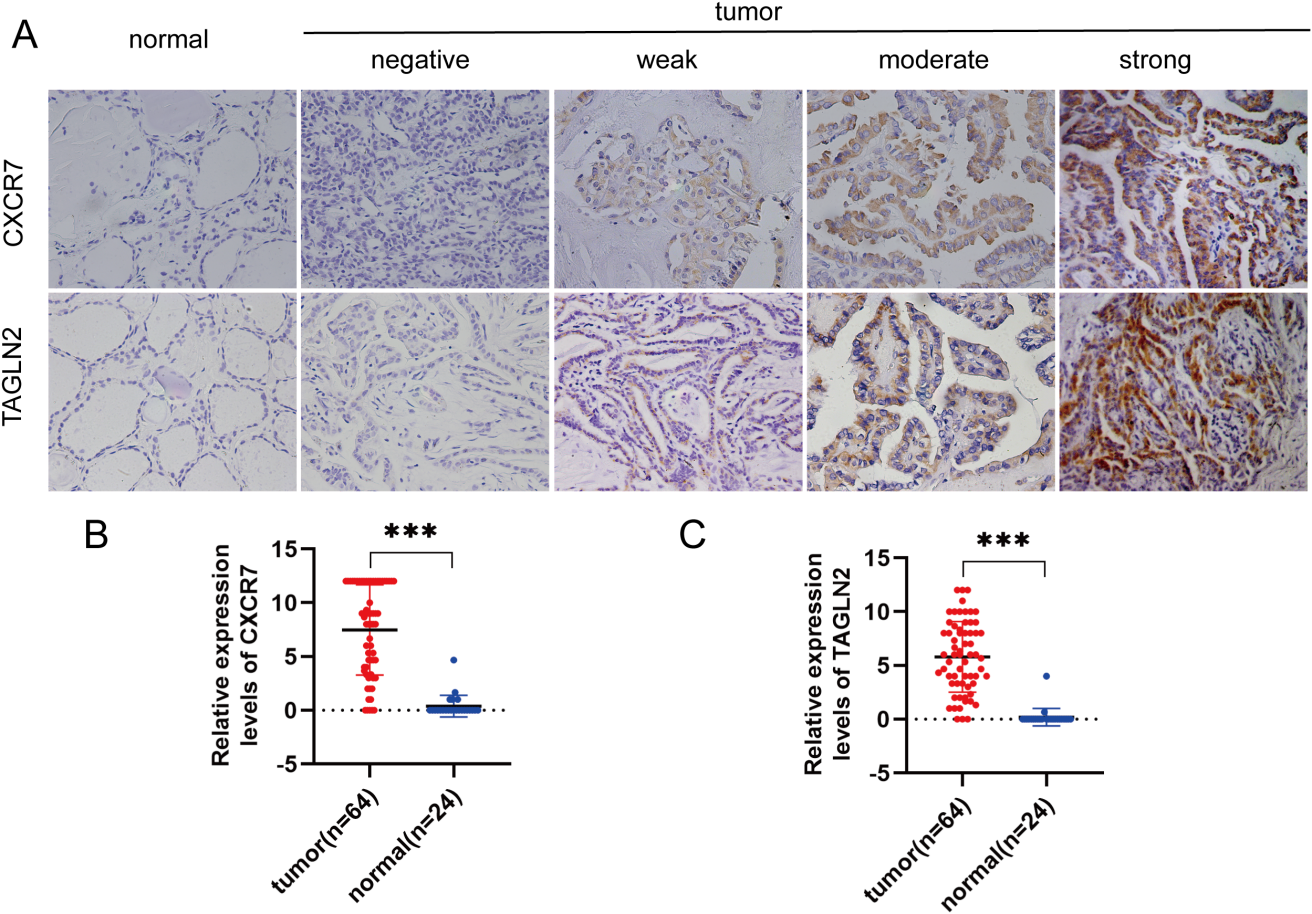
CXCR7 and TAGLN2 are highly expressed in PTC tissues. **(A)** Representative immunohistochemical staining (IHC) images of CXCR7 and TAGLN2 expressed in papillary thyroid carcinoma tissues and benign thyroid control tissues. **(B, C)** Bar charts of CXCR7 and TAGLN2 immunostaining scores. The relative expression levels of CXCR7 and TAGLN2 in PTC tumor tissues were significantly higher than those in adjacent normal tissues. *** indicates that p<0.001.

In PTC tissues, the positive expression rates of CXCR7 and TAGLN2 were 84.38% (54/64) and 79.69% (51/64), respectively, which were significantly higher than those observed in benign thyroid control tissues. Using Fisher’s exact test, the Odds Ratio for CXCR7 was 124.200 (95% CI: 15.016~1027.265, p < 0.001), and for TAGLN2 it was 90.231 (95% CI: 11.131~731.464, p < 0.001) (**Figure 1**).

To evaluate the clinical prognostic significance of CXCR7 and TAGLN2 in PTC, we performed Kaplan-Meier survival analysis on the TCGA thyroid papillary carcinoma (PTC) patient cohort. High expression of CXCR7 was significantly associated with poorer overall survival in PTC patients. The Kaplan-Meier survival curve revealed a statistically significant trend where patients with high CXCR7 expression exhibited a shorter overall survival compared to those with low CXCR7 expression (p=0.017) (**Supplementary Figure S1A**). In contrast, while TAGLN2 expression was significantly associated with lymph node metastasis in-house cohort, its association with overall patient survival in the TCGA cohort was not statistically significant (p=0.308) (**Supplementary Figure S1B**). This suggests that while TAGLN2 may contribute to tumor aggressiveness through invasion and metastasis, its direct prognostic value for overall survival in this dataset was not evident.

### 3.2 Correlation between the expressions of CXCR7 and TAGLN2 in PTC and clinicopathological characteristics

**Figure 2:**
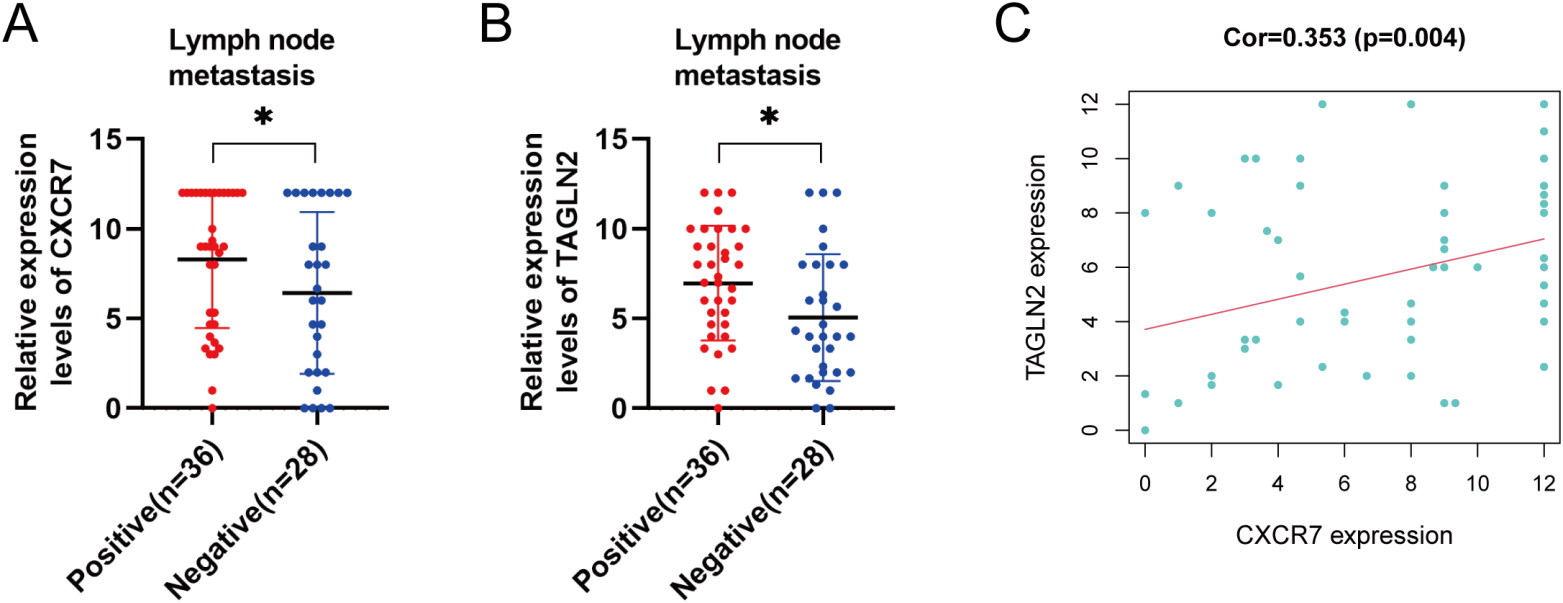
Correlation between the expressions of CXCR7 and TAGLN2 and clinicopathological characteristics. **(A, B)** The high expression of CXCR7 and TAGLN2 is closely related to lymph node metastasis of PTC. **(C)** CXCR7 is positively correlated with TAGLN2. Cor represents the correlation coefficient, and * indicates p<0.05.

We examined the association between CXCR7 and TAGLN2 expression levels and clinicopathological features of PTC. High expression of CXCR7 and TAGLN2 was significantly associated with lymph node metastasis (**Figures 2A, B**, p < 0.05), but not with other clinicopathological parameters, including age, sex, tumor size, capsular invasion, or multifocality (**Table 1**). High expression of CXCR7 and TAGLN2 was significantly associated with lymph node metastasis, as determined by Fisher’s exact test. For CXCR7, the Odds Ratio was 6.800 (95% CI: 1.313-35.230, p = 0.016), and for TAGLN2, it was 6.111 (95% CI: 1.489-25.087, p = 0.011). Furthermore, correlation analysis revealed a significant positive correlation between CXCR7 and TAGLN2 expression levels in PTC tissues (**Figure 2C**, R = 0.353, p = 0.004).

**Table 1:** Expression of CXCR7 and TAGLN2 in PTC tissues and their relationship with clinicopathological characteristics.

Clinicopathological characteristics	CXCR7 expression		P value	TAGLN2 expression		P value
	Positive (54)	Negative (10)		Positive (51)	Negative (13)	
Age			0.206			0.136
<55	44	6		42	8	
≥55	10	4		9	5	
Sex			1.000			1.000
Male	9	1		8	2	
Female	45	9		43	11	
Tumor size			1.000			0.507
≤ 2 cm	37	7		35	7	
>2cm,≤4cm	14	3		13	5	
>4cm	3	0		3	1	
Extrathyroid invasion			0.292			0.752
No	37	5		34	8	
Yes	17	5		17	5	
Lymph node metastasis			0.016			0.011
No	20	8		18	10	
Yes	34	2		33	3	
Multifocality			0.082			0.755
No	30	2		31	7	
Yes	24	8		20	6	

### 3.3 The co-immunoprecipitation experiment determined that intracellular CXCR7 and TAGLN2 had an interaction

**Figure 3:**
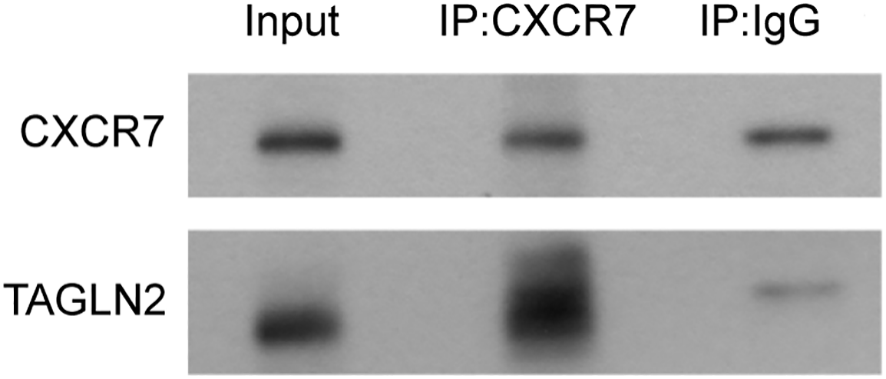
The interaction between CXCR7 and TAGLN2 detected by CP-IP. The experiment was divided into two groups: the Input group, the IP experimental group (IP: CXCR7), and the IP control group (IP: IgG). The IP: IgG group refers to the precipitation experiment conducted using IgG. The results showed that both CXCR7 and TAGLN2 were precipitated, indicating the possibility of non-specific binding between the two and the antibody. However, the non-specific binding of TAGLN2 was relatively weak. The IP: CXCR7 group refers to the precipitation experiment conducted using the CXCR7 protein. The results showed that both CXCR7 and TAGLN2 were precipitated, and the precipitation effect on TAGLN2 was very obvious. This indicates that there is an interaction between CXCR7 and TAGLN2.

To determine whether CXCR7 and TAGLN2 interact intracellularly, co-immunoprecipitation (Co-IP) experiments were performed. Total protein extracts from TPC-1 cells were immunoprecipitated using a CXCR7 antibody or IgG as a negative control. The resulting immune complexes were analyzed by Western blotting using antibodies against CXCR7 and TAGLN2. A markedly stronger TAGLN2 signal was detected in the CXCR7 immunoprecipitate compared to the IgG control (**Figure 3**), indicating that CXCR7 and TAGLN2 form a protein complex in PTC cells.

### 3.4 Immunofluorescence co-localization to determine the intracellular localization relationship between CXCR7 and TAGLN2 proteins

**Figure 4:**
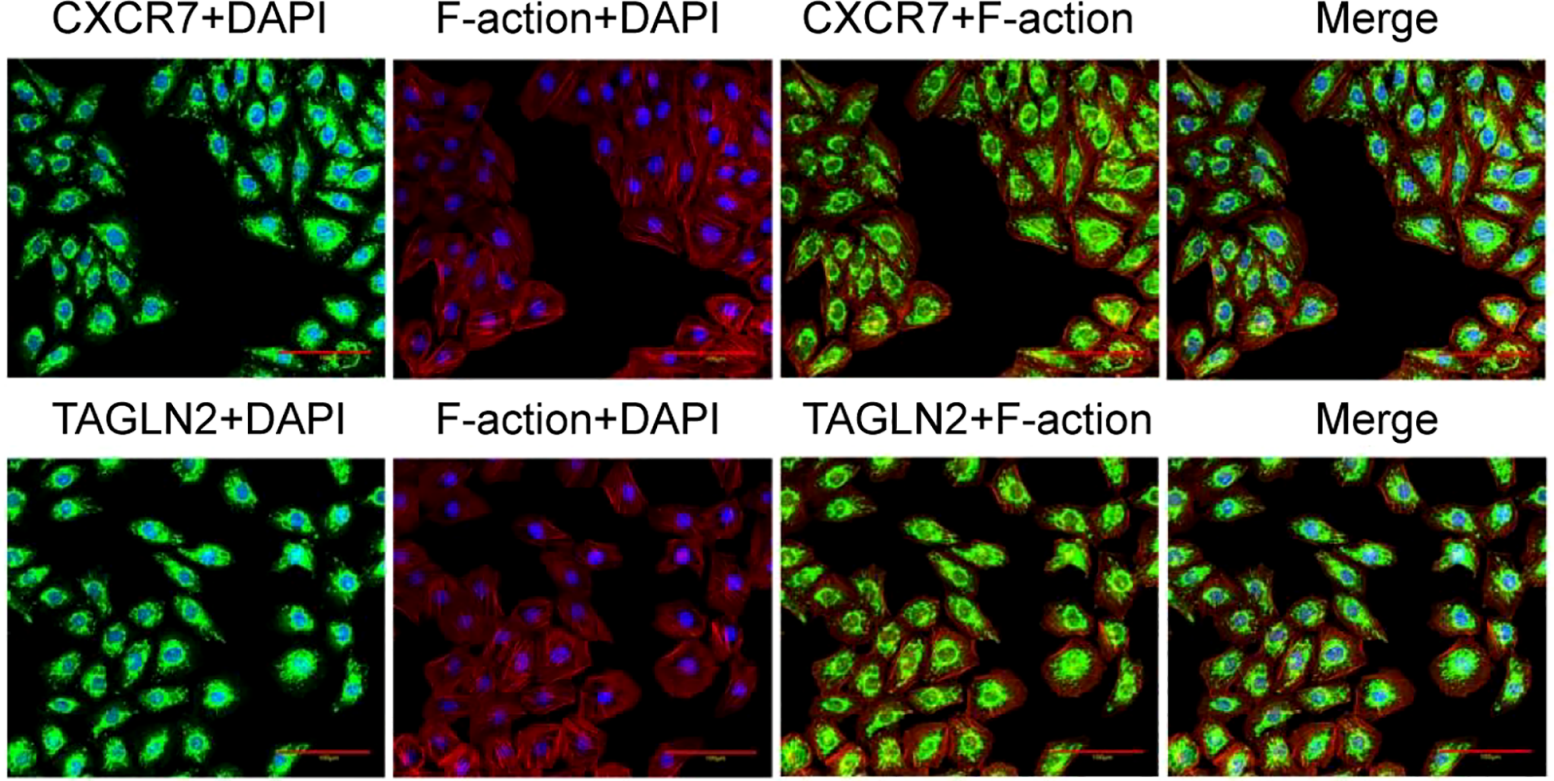
Co-localization of CXCR7 and TAGLN2 proteins with cytoskeleton immunofluorescence. The co-localization of CXCR7 (green) and TAGLN2 (green) with F-action (red) respectively. DAPI staining shows the cell nucleus (blue). Scale =100μm.

Immunofluorescence co-localization staining was conducted to assess the subcellular distribution of CXCR7 and TAGLN2. CXCR7 and TAGLN2 were labeled with green fluorescence, while the cytoskeleton was stained with rhodamine-labeled phalloidin. The fluorescence signals of CXCR7 and TAGLN2 exhibited a similar spatial distribution pattern within the cells (**Figure 4**), suggesting consistent subcellular localization of the two proteins.

### 3.5 To investigate the effects of co-transfection of “overexpression” CXCR7 and “silencing” TAGLN2 genes on the invasion and metastasis functions of PTC cells

**Figure 5:**
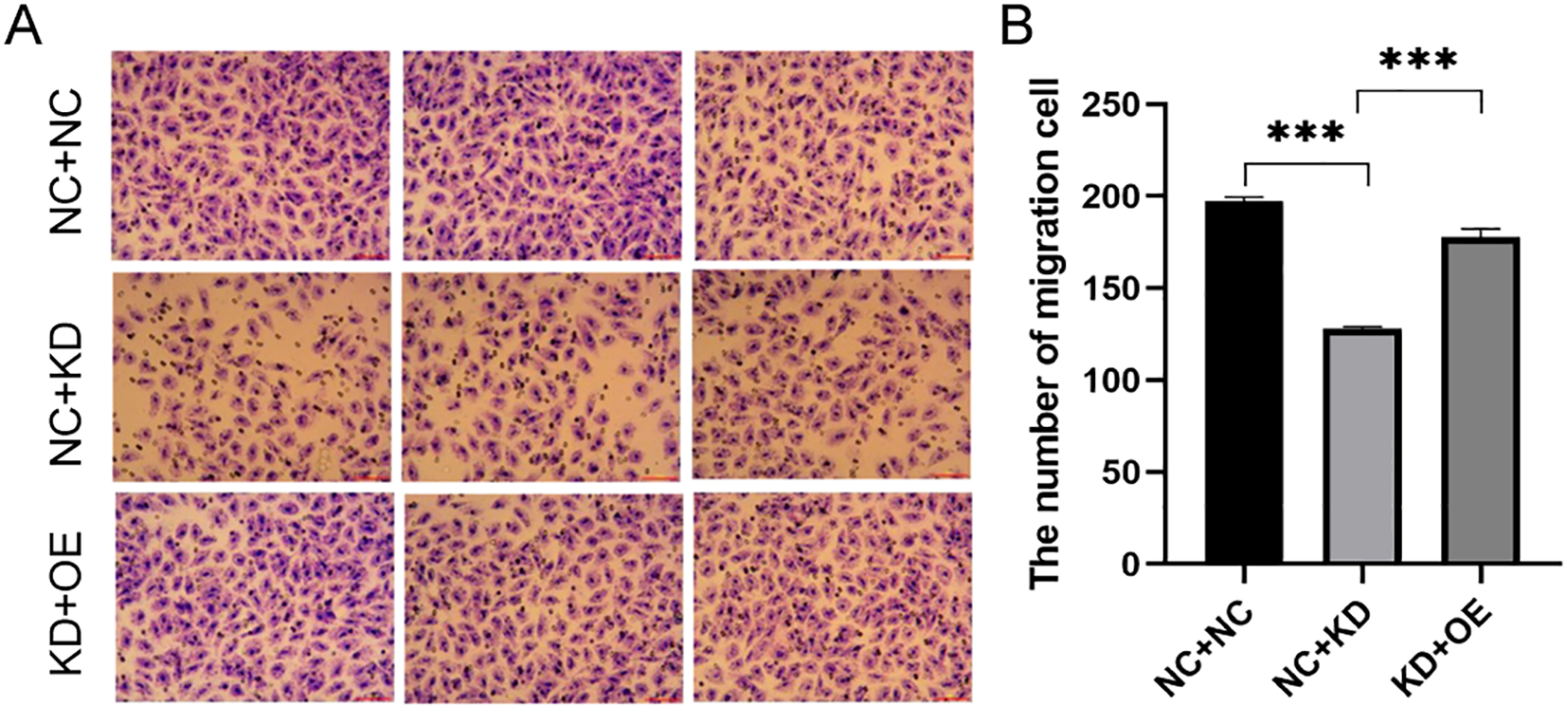
Effect of co-transfection of CXCR7 and TAGLN2 on the migration ability of TPC-1 cells. **(A)** Transwell migration assay was used to detect the effect of co-transfection of CXCR7 and TAGLN2 on the migration ability of TPC-1 cells; **(B)** The bar chart shows the number of migrated cells. NC+NC: No-load control group NC+KD: no-load control group +TAGLN2 knockdown group; KD+OE: TAGLN2 knockdown group + CXCR7 overexpression group. *** indicates that p<0.001.

To explore the functional interplay between CXCR7 and TAGLN2 in PTC, we established a stable TAGLN2-knockdown (KD) cell line and combined it with CXCR7 overexpression (OE). Migration Assays: Transwell migration assays were performed in both TPC-1 and BCPAP cell lines. In TPC-1 cells, silencing TAGLN2 significantly reduced cell migration compared to the empty vector control (NC+NC vs. NC+KD: 197.00 ± 2.082 vs. 128.00 ± 1.000, p < 0.001). Similarly, TAGLN2 knockdown in BCPAP cells resulted in a significant reduction in migration (NC+NC vs. NC+KD: 348 ± 42.5 vs. 153.3 ± 36, p = 0.001). Furthermore, introducing CXCR7 overexpression into the TAGLN2-knockdown background (OE+KD) significantly increased cell migration in both TPC-1 and BCPAP cells compared to the KD group alone (NC+KD vs KD+OE: TPC-1: 128.00 ± 1.000 vs. 178.00 ± 4.509, p < 0.001; BCPAP: 153.3 ± 36 vs. 266 ± 34.8, p = 0.011) (**Figure 5**, **Supplementary Figure S2**). These consistent findings across both TPC-1 and BCPAP cell lines strongly suggest that CXCR7 promotes PTC cell migration and invasion, potentially by counteracting the suppressive effects of TAGLN2.

**Figure 6:**
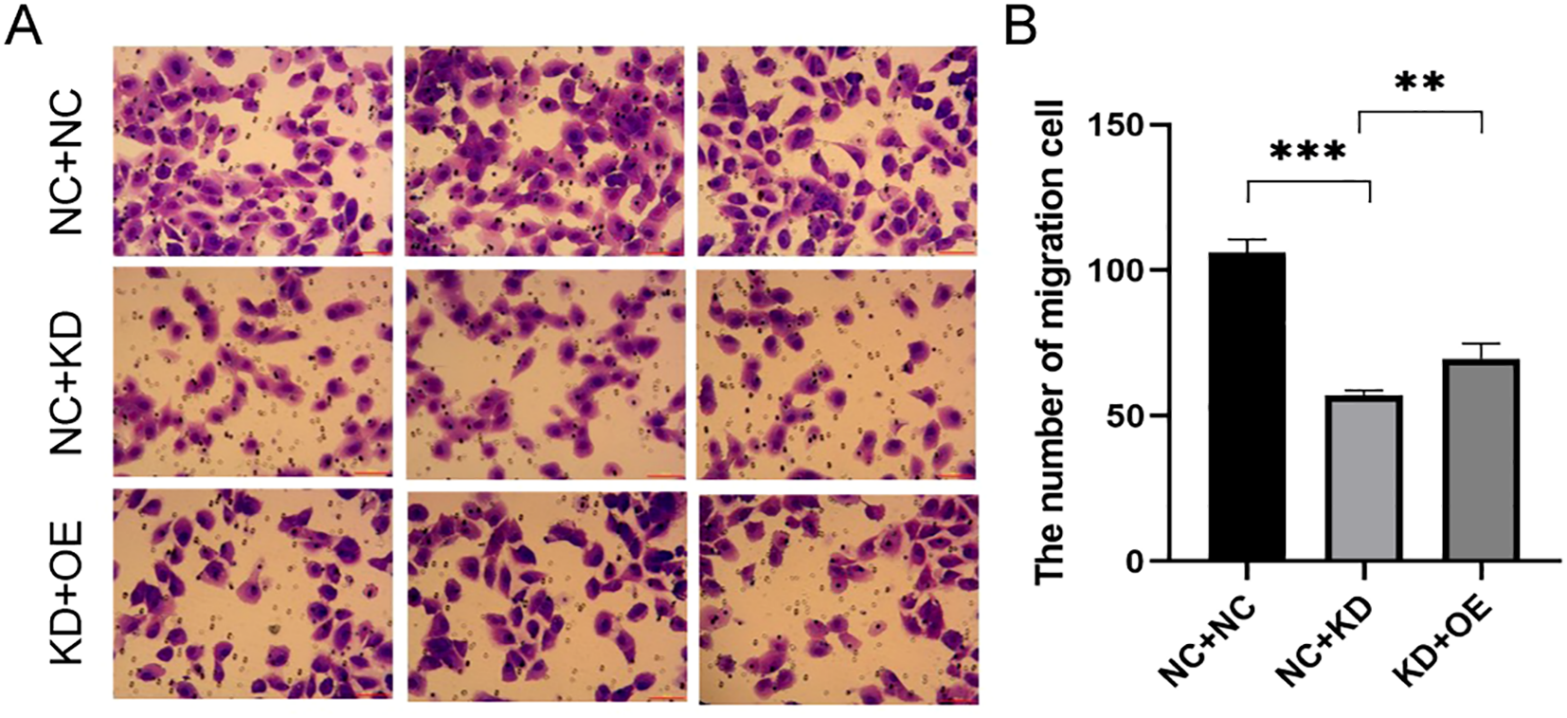
Effect of co-transfection of CXCR7 and TAGLN2 on the invasion ability of TPC-1 cells. **(A)** Transwell migration assay was used to detect the effect of co-transfection of CXCR7 and TAGLN2 on the migration ability of TPC-1 cells; **(B)** The bar chart shows the number of migrated cells. NC+NC: No-load control group NC+KD: no-load control group +TAGLN2 knockdown group; KD+OE: TAGLN2 knockdown group + CXCR7 overexpression group. ** indicates that p<0.01, *** indicates that p<0.001.

Invasion Assays: Next, we evaluated cell invasion. In TPC-1 cells, TAGLN2 silencing significantly reduced invasion compared to the control group (NC+NC vs. KD+NC: 106.00 ± 4.583 vs. 57.00 ± 1.732, p < 0.001). A similar observation was made in BCPAP cells, where TAGLN2 knockdown significantly reduced invasion (NC+NC vs. KD+NC: 285 ± 37.5 vs. 122 ± 12.5, p < 0.001). Upon co-expression of CXCR7 in the TAGLN2-silenced cells (KD+OE), cell invasion was significantly increased in both cell lines compared to the TAGLN2-silenced group alone (NC+KD vs KD+OE: TPC-1: 57.00 ± 1.732 vs. 70.00 ± 5.132, p = 0.009; BCPAP: 122 ± 12.5 vs. 226 ± 19.5, p = 0.009) (**Figure 6**, **Supplementary Figure S3**).

### 3.6 Bioinformatics analysis further evaluated the related signaling pathways of the interaction between CXCR7 and TAGLN2

**Figure 7:**
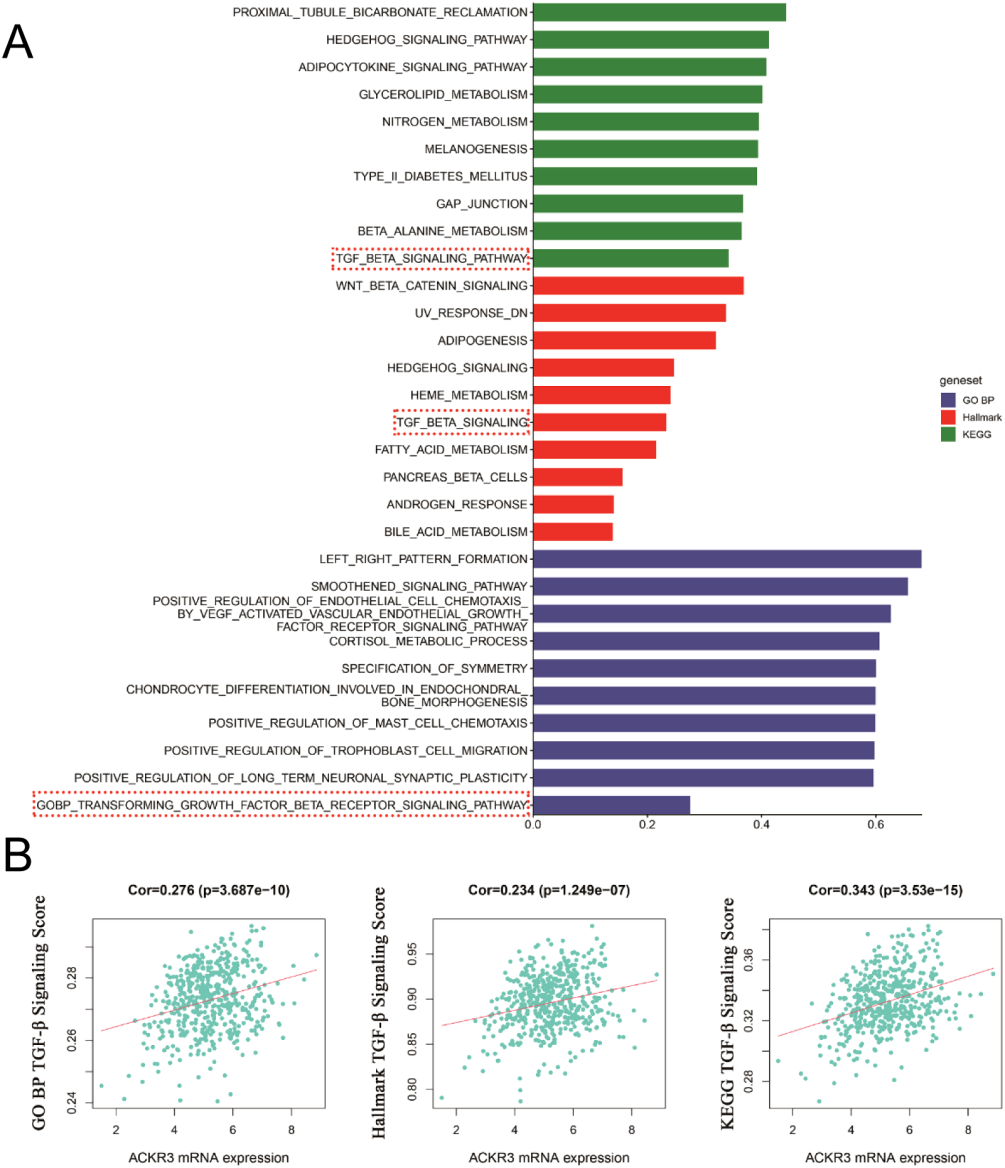
The ssGSEA score of the gene set in the TCGA database is correlated with the transcription level of CXCR7. **(A)** The top 10 signaling pathways with correlation coefficients between ssGSEA scores and CXCR7 transcriptional levels in Hallmark, GO, and KEGG gene sets. **(B)** The TGF-β signaling pathways in the Hallmark, GO (BP, biological process) and KEGG gene concentrations were linearly correlated with CXCR7. R represents the correlation coefficient, and p<0.05 indicates statistical significance.

**Figure 8:**
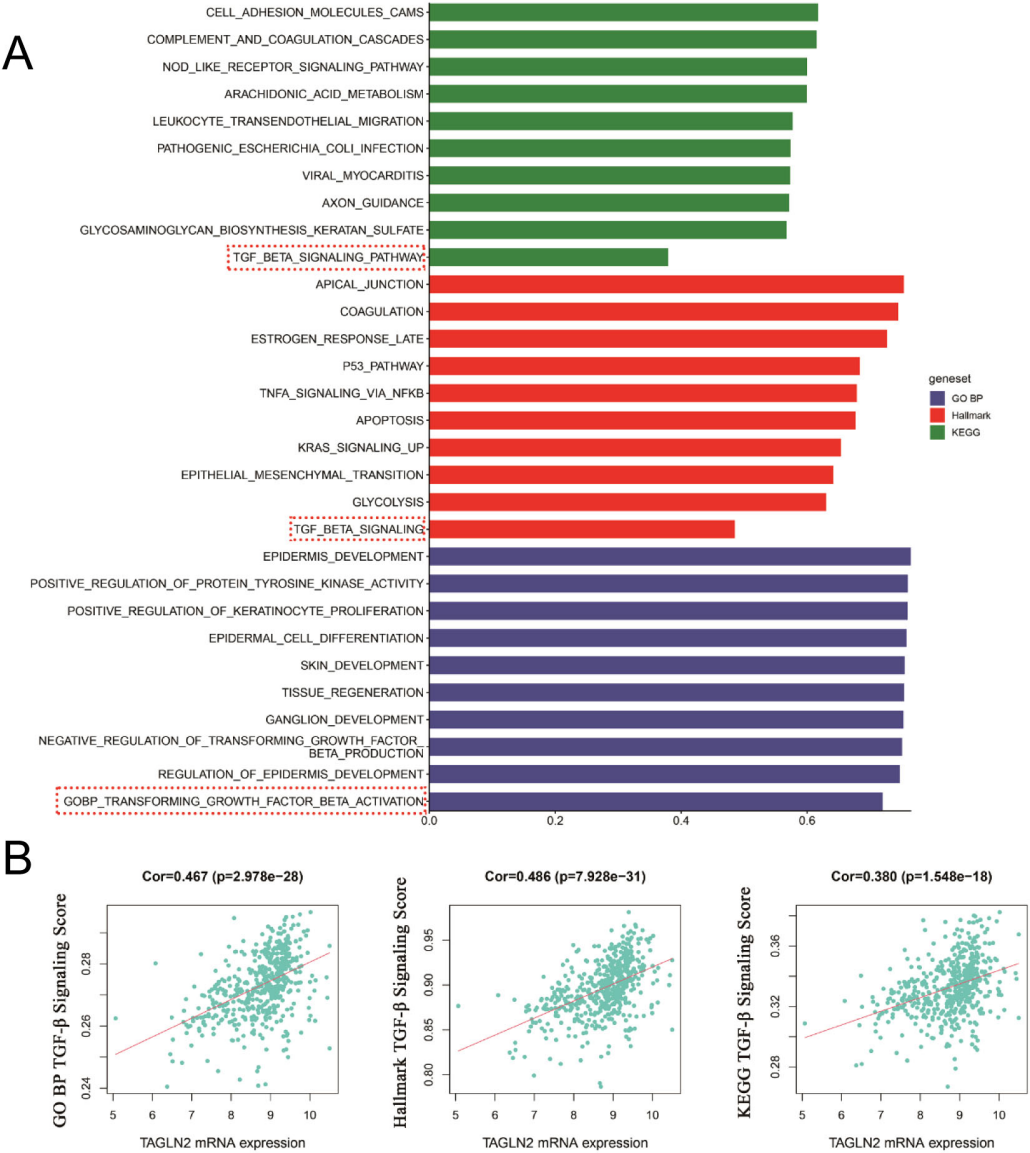
The ssGSEA score of the gene set in the TCGA database is correlated with the transcriptional level of TAGLN2. **(A)** The top 10 signaling pathways with correlation coefficients between ssGSEA scores in Hallmark, GO and KEGG gene sets and TAGLN2 transcriptional levels. **(B)** The TGF-β signaling pathways in the Hallmark, GO (BP, biological process) and KEGG gene concentrations were linearly correlated with TAGLN2. R represents the correlation coefficient, and p<0.05 indicates statistical significance.

We conducted a preliminary analysis of the biological determinants underlying CXCR7 and TAGLN2 expression in tumors through systematic big data mining. ssGSEA scores for the Hallmark, GO (biological process), and KEGG gene sets were calculated using sequencing data from 12 thyroid cancer cell lines in the CCLE database and 510 thyroid cancer cases from the TCGA database. Correlation analyses were then performed between the transcript abundance of CXCR7 and TAGLN2 and the ssGSEA scores of these gene sets. The results revealed that the TGF-β signaling pathway—across the Hallmark, GO (BP), and KEGG gene sets in the TCGA cohort—was positively correlated with the transcriptional levels of both CXCR7 and TAGLN2 (**Figures 7**, **8**), suggesting that the biological effects of CXCR7 and TAGLN2 may be modulated by the TGF-β signaling pathway.

### 3.7 CXCR7/TAGLN2-TGF-β/Smad2 axis promotes PTC aggressiveness

Our findings elucidate a novel mechanism by which CXCR7 and TAGLN2 cooperate to promote PTC progression, primarily through the canonical TGF-β/Smad2 signaling pathway. Functional assays in both TPC-1 and BCPAP cell lines consistently demonstrated that silencing TAGLN2 significantly attenuated cell migration and invasion, highlighting TAGLN2’s role in supporting these aggressive phenotypes. Critically, the re-expression of CXCR7 in TAGLN2-knockdown cells rescued these suppressive effects, indicating that CXCR7 promotes migration and invasion, possibly by counteracting TAGLN2’s inhibitory influence. Mechanistically, these functional observations were strongly supported by Western blot analysis. In both cell lines, TAGLN2 knockdown led to a marked decrease in phosphorylated Smad2 (p-Smad2) levels, confirming TAGLN2’s contribution to the basal activation of the TGF-β signaling pathway. Furthermore, re-introducing CXCR7 into TAGLN2-silenced cells restored p-Smad2 levels, demonstrating that CXCR7 actively promotes TGF-β/Smad2 signaling (**Supplementary Figure S4**). Thus, we establish that the functional axis of CXCR7 and TAGLN2, acting through the activation of the Smad2 pathway, is a critical driver of PTC aggressiveness, a mechanism consistently validated across two distinct PTC cell models.

## 4 Discussion

Chemokines, a family of small secreted proteins, regulate cell migration by binding to specific receptors, forming a complex network that influences tumor development. Accumulating evidence underscores the critical role of chemokines and their receptors in tumorigenesis, particularly in directing the migration of cancer cells from primary sites to distant organs. Within the tumor microenvironment (TME), chemokines produced by both tumor and stromal cells act on target cells to modulate the TME, drive tumor-specific immune responses, and promote tumor invasion, metastasis, angiogenesis, and resistance to chemotherapy and radiotherapy (20, 21).

The chemokine system, particularly the SDF-1/CXCR7 axis, plays a pivotal role in orchestrating the tumor microenvironment and regulating cancer cell behavior. SDF-1, a potent chemoattractant for lymphocytes, is aberrantly expressed in thyroid cancer, contributing to tumorigenesis and serving as a potential prognostic biomarker (22). As a scavenger receptor for SDF-1, CXCR7 fine-tunes the chemokine gradient within the TME, thereby influencing directional migration of tumor cells and potentially modulating immune cell infiltration (23). Our previous studies have established CXCR7 as a key promoter of PTC development, invasion, and metastasis (11, 12), highlighting its significance in disease progression. To further elucidate the molecular mechanisms underlying CXCR7-mediated effects, we employed a comprehensive proteomic approach using iTRAQ coupled with 2DLC-MS/MS to profile protein expression changes in CXCR7-overexpressing PTC cells relative to untransfected controls (19). This unbiased analysis identified several significantly upregulated proteins, including FN1, BSG, PPL, SERPINB5, TAGLN2, and CD147. Among these, TAGLN2 was of particular interest due to its marked upregulation upon CXCR7 overexpression and the limited understanding of its role in PTC metastasis. This observation led us to investigate TAGLN2 as a potential mediator of CXCR7’s pro-metastatic effects and a candidate therapeutic target.

TAGLN2, initially identified as a cytoskeletal protein involved in the stabilization of actin stress fibers, has emerged as a multifunctional regulator in cancer biology, contributing to various processes that drive tumorigenesis and disease progression. Elevated TAGLN2 expression has been frequently observed across multiple malignancies, including bladder cancer (24), pancreatic cancer (25), colorectal cancer (26), renal cell carcinoma (27), esophageal cancer (28), cholangiocarcinoma (28), and gastric cancer (29), and is associated with enhanced tumor cell proliferation, invasion, metastasis, and epithelial-mesenchymal transition (EMT). These effects are largely mediated through the modulation of key signaling pathways, underscoring TAGLN2’s ability to integrate diverse cellular signals and coordinate complex cellular responses. In PTC, TAGLN2 is also highly expressed and promotes cell migration and invasion, at least partially via activation of the Rap1/PI3K/AKT signaling pathway (18). While these findings highlight the significance of TAGLN2 in PTC progression, the interaction between CXCR7 and TAGLN2, as well as the detailed molecular mechanisms underlying their potential synergistic role in regulating PTC invasion and metastasis, remains poorly understood. This gap in knowledge presents a critical avenue for future research aimed at elucidating the molecular networks driving PTC metastasis and identifying novel therapeutic targets.

Our initial analysis focused on characterizing the expression patterns of CXCR7 and TAGLN2 in PTC tissues, revealing significant upregulation of both proteins compared to adjacent non-tumor thyroid tissues (CXCR7, p < 0.001; TAGLN2, p < 0.001). Furthermore, elevated expression levels of CXCR7 and TAGLN2 were significantly associated with lymph node metastasis in PTC patients (CXCR7, p < 0.05; TAGLN2, p < 0.05; R = 0.353), providing strong evidence for their involvement in PTC progression. Given that lymph node metastasis is a well-established predictor of adverse prognosis in PTC, these findings underscore the clinical relevance of CXCR7 and TAGLN2 and suggest their potential utility as prognostic biomarkers. The coordinated overexpression of CXCR7 and TAGLN2, along with their association with metastatic disease, implies a synergistic role in promoting PTC invasion and metastasis, rather than independent contributions.

The significant association of CXCR7 and TAGLN2 with lymph node metastasis underscores their clinical relevance in the context of aggressive PTC. To further investigate their prognostic value, we performed Kaplan-Meier survival analysis using TCGA PTC patient data. Our analysis revealed that high expression of CXCR7 was significantly correlated with poorer overall survival (p = 0.017), indicating that elevated CXCR7 levels predict a worse clinical outcome in PTC patients. This finding is in line with the pro-invasive and pro-metastatic roles of CXCR7 observed in our *in vitro* studies and highlights its potential as a prognostic biomarker for high-risk PTC. In contrast, our survival analysis did not identify a statistically significant correlation between TAGLN2 expression levels and overall patient survival (p = 0.308). While TAGLN2 has been mechanistically linked to cellular invasion, its direct prognostic value for overall survival in this TCGA cohort was not significant. This observation might be attributed to several factors: firstly, TAGLN2’s primary role could be in promoting earlier stages of tumor progression, such as invasion and dissemination to lymph nodes, as indicated by its correlation with metastasis. Its direct impact on long-term overall survival might be less pronounced or influenced by other factors. Secondly, given the generally favorable prognosis of PTC, identifying prognostic markers that significantly impact overall survival can be challenging, and the effect of TAGLN2 might be more subtle, context-dependent, or manifest within specific patient subgroups. Further studies, potentially with larger, stratified cohorts or by analyzing TAGLN2 in combination with other markers, might be necessary to fully elucidate its prognostic significance.

To explore the mechanistic basis of the association between CXCR7 and TAGLN2 in PTC metastasis, we investigated the possibility of a direct protein-protein interaction. Immunofluorescence co-localization analysis demonstrated spatial proximity of CXCR7 and TAGLN2 within PTC cells, suggesting potential physical interaction. This observation was confirmed by co-immunoprecipitation assays, which verified the formation of protein complexes containing both CXCR7 and TAGLN2 in PTC cells. The identification of a direct molecular interaction between CXCR7 and TAGLN2 establishes a critical mechanistic link, indicating that these proteins may function as a complex to regulate cellular processes underlying PTC invasion and metastasis. Such an interaction may modulate the signaling properties of both proteins, leading to amplified activation of downstream pathways that enhance cell migration, invasion, and survival. These findings provide a foundation for future studies to characterize the structural and functional features of the CXCR7-TAGLN2 complex and to develop targeted strategies aimed at disrupting its formation or activity, potentially offering improved therapeutic approaches for PTC.

TGF-β signal transduction serves as a central regulator of tumor progression, exerting pleiotropic effects on cancer cells that collectively promote malignant behavior. Through autocrine and paracrine signaling mechanisms, TGF-β modulates epithelial cell phenotype, enhances tumor cell invasion and dissemination, promotes stem cell-like properties, and contributes to chemoresistance (30). Given the well-documented role of TGF-β in cancer pathogenesis, we explored its potential to regulate the coordinated activities of CXCR7 and TAGLN2 in PTC. The clinical significance of this hypothesis is supported by findings from Zhang et al., who demonstrated significantly elevated expression levels of TGF-β1 and phosphorylated Smad3 (p-Smad3) in PTC tissues compared to normal thyroid tissue, with TGF-β1 expression showing a strong positive correlation with lymph node metastasis (31, 32). These data indicate that TGF-β signaling plays a pivotal role in driving aggressive features of PTC. Together with existing evidence linking TGF-β signaling to both TAGLN2 and CXCR7 regulation, these observations led us to hypothesize that TGF-β may act as a key upstream modulator of the CXCR7-TAGLN2 axis, coordinately regulating their expression and function to facilitate PTC invasion and metastasis. This notion is reinforced by previous studies showing that TGF-β upregulates TAGLN2 expression in human adipocytes (33), promotes Smad4-dependent vascular invasion in hepatocellular carcinoma (34), and directly activates TAGLN2 transcription via the TGF-β/Smad4 pathway in colon cancer cells (35). Additionally, CXCR7 function is subject to modulation by TGF-β signaling, as knockdown of CXCR7 has been shown to attenuate TGF-β1-induced migration, invasion, and epithelial-mesenchymal transition (EMT) in lung cancer cells (36), whereas CXCR7 overexpression enhances TGF-β1 secretion and promotes EMT through Smad2/3 phosphorylation in head and neck squamous cell carcinoma (37). Supporting our hypothesis, bioinformatics analysis revealed a significant positive correlation between TGF-β signaling activity and the mRNA expression levels of both CXCR7 and TAGLN2 in PTC tissues, further suggesting that TGF-β signaling may serve as a central driver of CXCR7-TAGLN2-mediated invasion and metastasis in PTC. Crucially, our experiments revealed that knockdown of TAGLN2 in thyroid papillary carcinoma cells led to a significant decrease in p-Smad2 levels, indicating an attenuation of TGF-β signaling activation. This suggests that TAGLN2 may play a role in promoting or sustaining TGF-β pathway activity. Furthermore, in a compelling rescue experiment, overexpression of CXCR7 upon TAGLN2 knockdown restored p-Smad2 levels to near-baseline, implying a cooperative interaction between TAGLN2 and CXCR7 in modulating TGF-β signaling. Importantly, total Smad2 protein levels remained unchanged across all experimental conditions, confirming that these effects are due to pathway activation rather than changes in protein abundance. This direct evidence establishes a functional link between the TAGLN2-CXCR7 axis and the modulation of TGF-β signaling, suggesting that this axis may be a key upstream regulator or a critical component in the activation cascade of TGF-β in PTC.

While our current study strongly implicates TGF-β signaling in the coordinated regulation of CXCR7 and TAGLN2 in PTC invasion, we acknowledge that other signaling pathways may also contribute to these processes. Specifically, in our previous work, we have identified alternative pathways such as the Rap1/PI3K/AKT signaling axis as crucial regulators of thyroid cancer cell invasion and metastasis (18). These pathways are known to crosstalk with TGF-β signaling or regulate similar cellular processes independently. For instance, the PI3K/AKT pathway is a key mediator of cell proliferation, survival, and migration, and has been implicated in regulating EMT and chemoresistance in various cancers, including thyroid cancer (38). Furthermore, Rap1, a small GTPase, has been shown to influence cell adhesion, migration, and invasion (39), and its downstream effectors can overlap with those regulated by TGF-β. Future studies are warranted to comprehensively investigate the interplay between TGF-β, Rap1/PI3K/AKT signaling, and the CXCR7-TAGLN2 axis in PTC tumorigenesis.

In summary, this study provides robust evidence implicating the CXCR7-TAGLN2 axis in PTC progression. Our results demonstrate that both CXCR7 and TAGLN2 are markedly overexpressed in PTC tissues, and high expression levels of these proteins are strongly associated with lymph node metastasis, a key prognostic indicator. Furthermore, we show that CXCR7 and TAGLN2 physically interact within PTC cells, forming a protein complex that appears to play a critical role in regulating tumor cell invasion and metastasis. This interaction is likely influenced, at least in part, by TGF-β signaling, positioning the TGF-β–CXCR7/TAGLN2 signaling cascade as a potential target for early diagnosis and therapeutic intervention in PTC.

While these findings represent an important advance in understanding PTC biology, several limitations exist. A primary limitation of our study lies in the relatively small size of our initial, retrospective clinical cohort (64 PTC cases and 24 benign controls). Stratification by adverse features, such as lymph node metastasis, further reduced the subgroup size, which consequently limits statistical power and increases the potential for selection bias. We have mitigated this by employing the Fisher’s Exact Test for categorical comparisons and consistently reporting effect sizes with 95% Confidence Intervals. Crucially, the findings derived from this small cohort, particularly the prognostic significance of CXCR7, have been externally validated using the comprehensive TCGA PTC dataset. This external validation substantially enhances the overall reliability and clinical relevance of our observations. Future studies should aim to validate these findings prospectively in a larger, independent patient cohort to confirm generalizability definitively. Furthermore, the *in vitro* experimental findings, including Western blot analyses, provide strong evidence for the involvement of the TGF-β pathway in regulating the CXCR7/TAGLN2 complex and promoting PTC progression. However, our current study lacks *in vivo* validation, which is crucial for establishing the translational relevance of these interactions under physiologically relevant conditions. While our Western blot experiments have confirmed the functional role of the TGF-β pathway in response to genetic manipulations of CXCR7 and TAGLN2 in cell lines, comprehensive *in vivo* studies, such as xenograft or metastasis models, are essential to elucidate how this axis impacts tumor growth, invasion, and metastatic potential within a complex biological microenvironment. Moreover, future studies should employ *in vivo* models to evaluate the therapeutic efficacy of targeting this interaction, thus paving the way for its translation into clinically applicable strategies for the management of PTC.

## Data Availability

The original contributions presented in the study are included in the article/**Supplementary Material**. Further inquiries can be directed to the corresponding author.

## Supplementary material

Supplementary Figure 1 Kaplan-Meier survival analysis of CXCR7 and TAGLN2 expression in PTC patients from the TCGA database. **(A)** Overall survival curves stratified by CXCR7 expression level. Patients were categorized into high (red line) and low (blue line) expression groups based on the median expression value. High CXCR7 expression was significantly associated with shorter overall survival (p=0.017). **(B)** Overall survival curves stratified by TAGLN2 expression level. Patients were categorized into high (red line) and low (blue line) expression groups based on the median expression value. No significant difference in overall survival was observed between the high and low TAGLN2 expression groups (p=0.308). The log-rank test was used for comparison.

Supplementary Figure 2 Effect of co-transfection of CXCR7 and TAGLN2 on the migration ability of BCPAP cells. **(A)** Transwell migration assay was used to detect the effect of co-transfection of CXCR7 and TAGLN2 on the migration ability of BCPAP cells; **(B)** The bar chart shows the number of migrated cells. NC+NC: No-load control group NC+KD: no-load control group +TAGLN2 knockdown group; KD+OE: TAGLN2 knockdown group + CXCR7 overexpression group. * indicates that p<0.05, ** indicates that p<0.01.

Supplementary Figure 3 Effect of co-transfection of CXCR7 and TAGLN2 on the invasion ability of BCPAP cells. **(A)** Transwell migration assay was used to detect the effect of co-transfection of CXCR7 and TAGLN2 on the migration ability of BCPAP cells; **(B)** The bar chart shows the number of migrated cells. NC+NC: No-load control group NC+KD: no-load control group +TAGLN2 knockdown group; KD+OE: TAGLN2 knockdown group + CXCR7 overexpression group. ** indicates that p<0.01, *** indicates that p<0.001.

Supplementary Figure 4 Western blot analysis of Smad2 and p-Smad2 expression in TPC-1 and BCPAP cells. **(A)** Representative Western blot images showing the expression levels of p-Smad2, total Smad2, and GAPDH in control, TAGLN2-silenced (NC+KD), and TAGLN2-silenced + CXCR7-overexpressed (KD+OE) TPC-1 and BCPAP cells. **(B)** Bar graph quantifying the relative expression levels of total Smad2 in TPC-1 cells under the different treatment conditions. **(C)** Bar graph quantifying the relative expression levels of total Smad2 in BCPAP cells under the different treatment conditions. **(D)** Bar graph quantifying the relative expression levels of p-Smad2 in TPC-1 cells under the different treatment conditions. **(E)** Bar graph quantifying the relative expression levels of p-Smad2 in BCPAP cells under the different treatment conditions. Data are presented as mean ± SD from three independent experiments. * indicates that p<0.05, ** indicates that p<0.01, ns indicates no significance. GAPDH was used as an internal loading control.
