# Supplementary figures and images for: CXCR7-TAGLN2 protein complex regulates invasion and metastasis in papillary thyroid carcinoma: a potential therapeutic target
Source: Front Immunol. 2025 Oct 15;16:1627419. doi: 10.3389/fimmu.2025.1627419 (PMC12568504; doi:10.3389/fimmu.2025.1627419)

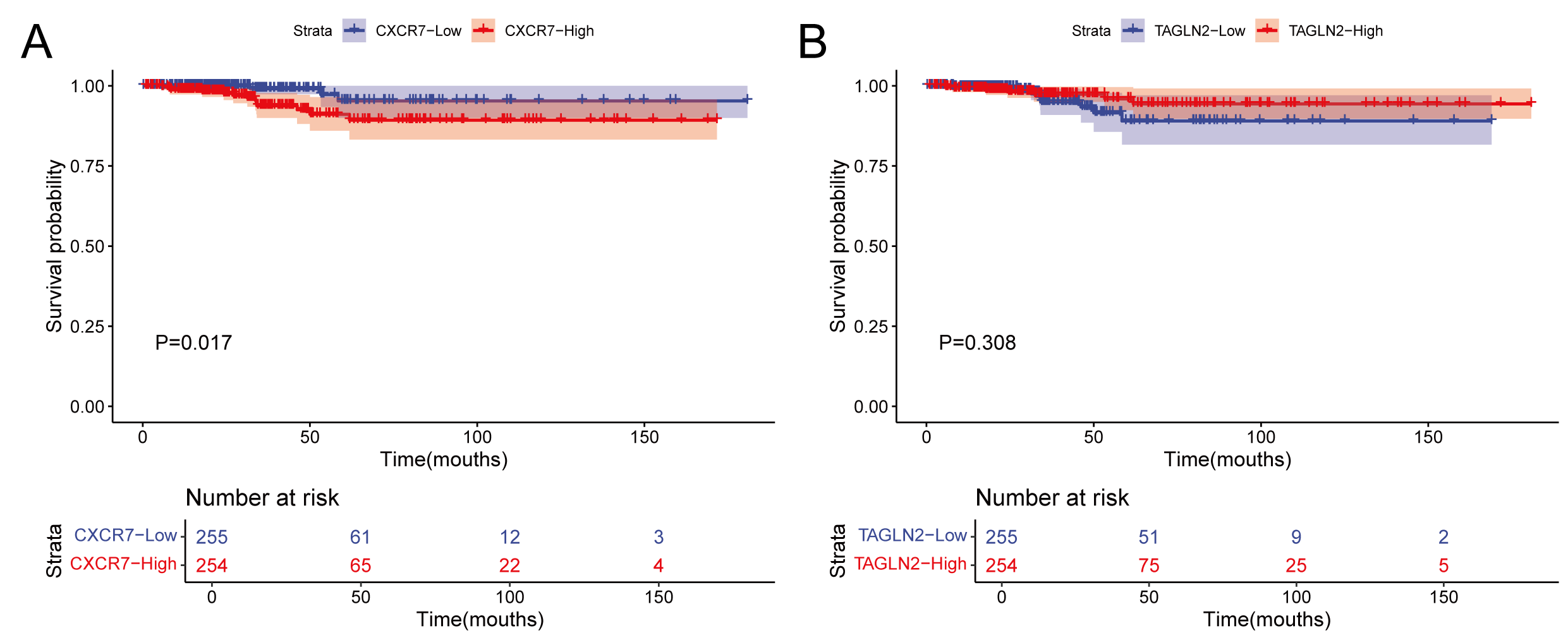

Supplement: Supplementary Figure 1 — Kaplan-Meier survival analysis of CXCR7 and TAGLN2 expression in PTC patients from the TCGA database. (A) Overall survival curves stratified by CXCR7 expression level. Patients were categorized into high (red line) and low (blue line) expression groups based on the median expression value. High CXCR7 expression was significantly associated with shorter overall survival (p=0.017). (B) Overall survival curves stratified by TAGLN2 expression level. Patients were categorized into high (red line) and low (blue line) expression groups based on the median expression value. No significant difference in overall survival was observed between the high and low TAGLN2 expression groups (p=0.308). The log-rank test was used for comparison. [file Image1.tif]

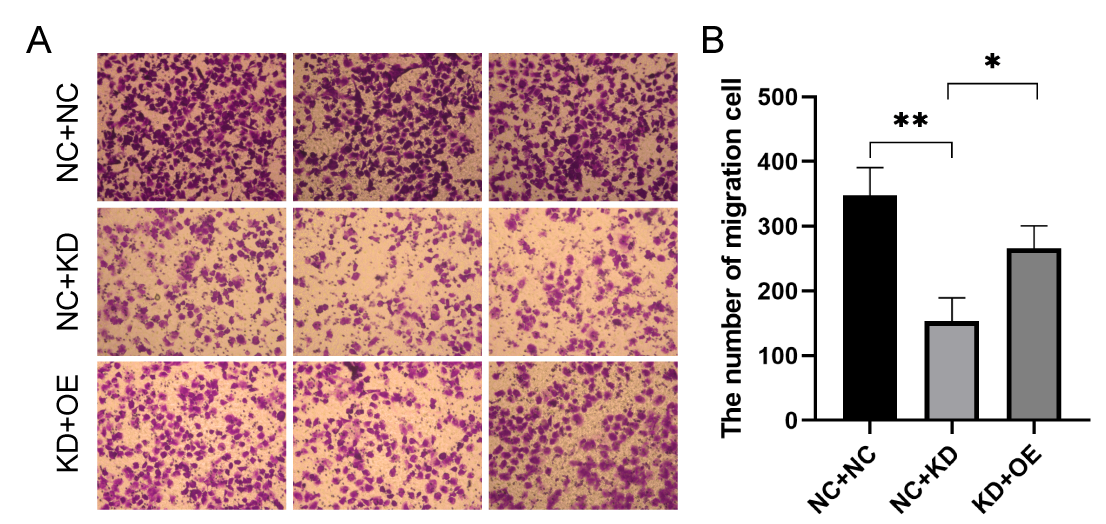

Supplement: Supplementary Figure 2 — Effect of co-transfection of CXCR7 and TAGLN2 on the migration ability of BCPAP cells. (A) Transwell migration assay was used to detect the effect of co-transfection of CXCR7 and TAGLN2 on the migration ability of BCPAP cells; (B) The bar chart shows the number of migrated cells. NC+NC: No-load control group NC+KD: no-load control group +TAGLN2 knockdown group; KD+OE: TAGLN2 knockdown group + CXCR7 overexpression group. * indicates that p<0.05, ** indicates that p<0.01. [file Image2.tif]

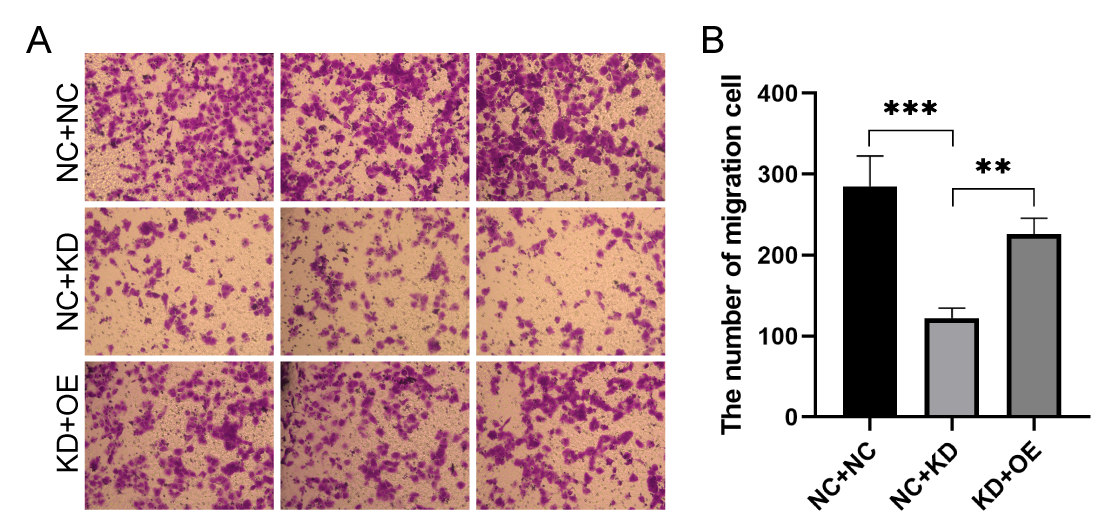

Supplement: Supplementary Figure 3 — Effect of co-transfection of CXCR7 and TAGLN2 on the invasion ability of BCPAP cells. (A) Transwell migration assay was used to detect the effect of co-transfection of CXCR7 and TAGLN2 on the migration ability of BCPAP cells; (B) The bar chart shows the number of migrated cells. NC+NC: No-load control group NC+KD: no-load control group +TAGLN2 knockdown group; KD+OE: TAGLN2 knockdown group + CXCR7 overexpression group. ** indicates that p<0.01, *** indicates that p<0.001. [file Image3.tif]

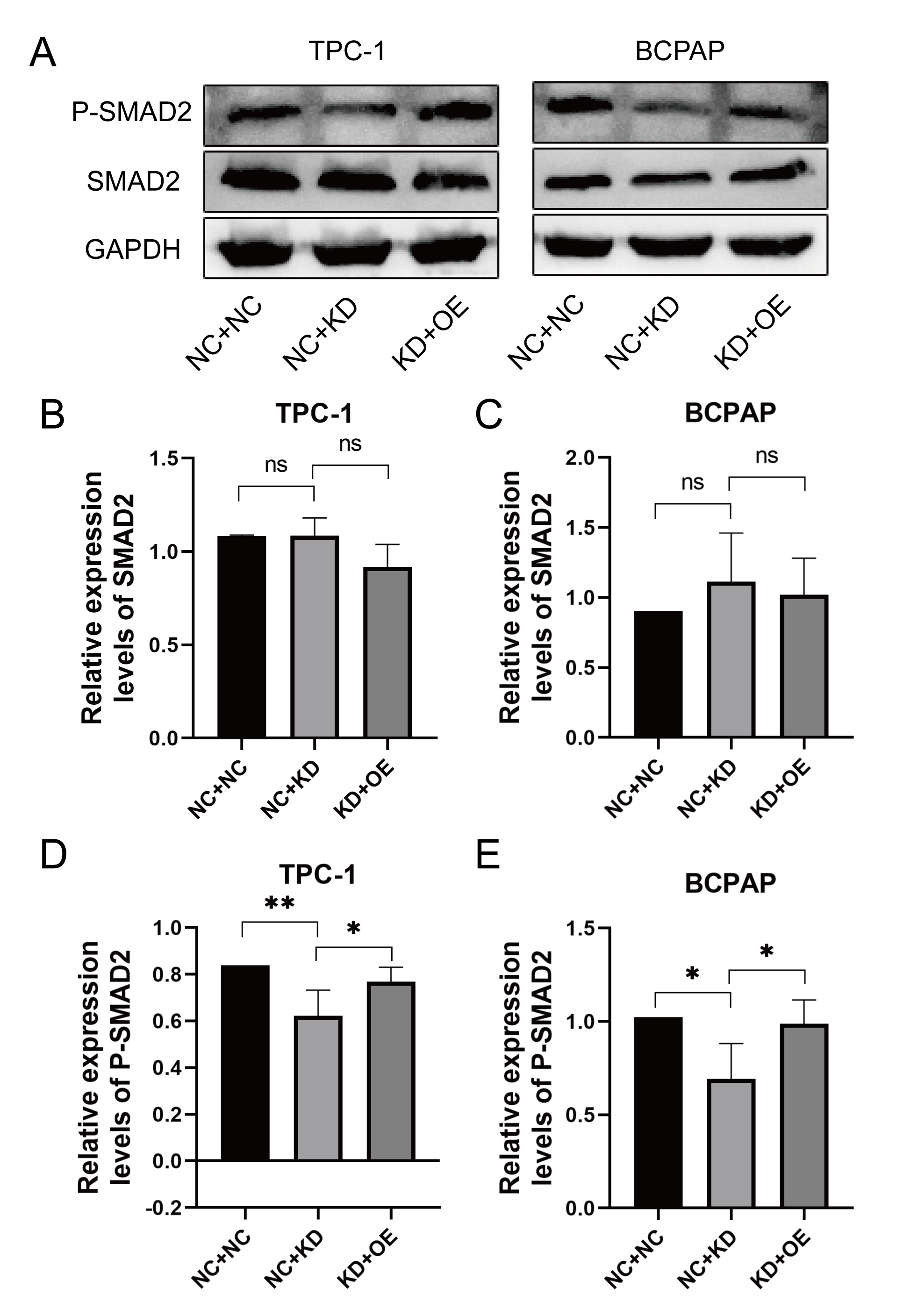

Supplement: Supplementary Figure 4 — Western blot analysis of Smad2 and p-Smad2 expression in TPC-1 and BCPAP cells. (A) Representative Western blot images showing the expression levels of p-Smad2, total Smad2, and GAPDH in control, TAGLN2-silenced (NC+KD), and TAGLN2-silenced + CXCR7-overexpressed (KD+OE) TPC-1 and BCPAP cells. (B) Bar graph quantifying the relative expression levels of total Smad2 in TPC-1 cells under the different treatment conditions. (C) Bar graph quantifying the relative expression levels of total Smad2 in BCPAP cells under the different treatment conditions. (D) Bar graph quantifying the relative expression levels of p-Smad2 in TPC-1 cells under the different treatment conditions. (E) Bar graph quantifying the relative expression levels of p-Smad2 in BCPAP cells under the different treatment conditions. Data are presented as mean ± SD from three independent experiments. * indicates that p<0.05, ** indicates that p<0.01, ns indicates no significance. GAPDH was used as an internal loading control. [file Image4.tif]
